# Physical Performance Indicators and Team Success in the German Soccer League

**DOI:** 10.2478/hukin-2022-0099

**Published:** 2022-09-08

**Authors:** Paweł Chmura, José M. Oliva-Lozano, José M. Muyor, Marcin Andrzejewski, Jan Chmura, Sławomir Czarniecki, Edward Kowalczuk, Andrzej Rokita, Marek Konefał

**Affiliations:** 1 Department of Team Games, Wrocław University of Health and Sport Sciences, 51-612 Wrocław, Poland; 2 Health Research Centre, University of Almería, Almería, Spain; 3 Laboratory of Kinesiology, Biomechanics and Ergonomics (KIBIOMER Lab), Research Central Services, University of Almería, Almería, Spain; 4 Department of Methodology of Recreation, Poznań University of Physical Education, 61-871 Poznań, Poland; 5 Department of Biological and Motor Sport Bases, Wrocław University of Health and Sport Sciences, 51-612 Wrocław, Poland; 6 Football Club, Bayer 04 Leverkusen, 51373 Leverkusen, Germany; 7 Football Club, Hannover 96, Hannover 30169, Germany

**Keywords:** match analysis, tracking systems, external load, monitoring, European football

## Abstract

The aims of this study were (1) to determine the match running performance required by different teams based on their final ranking position and (2) to analyze the association between match running performance variables and team success at the end of the season. A total of 1,224 match observations from professional soccer teams competing during two consecutive seasons in the German Bundesliga were analyzed. In addition, the final league ranking position and the total of points obtained by each team at the end of the season were registered for the analysis of the association between team success and match running performance. The main findings were that high ranked teams covered the greatest total distance with ball possession, sprinting distance with ball possession, and completed the greatest number of sprinting actions with ball possession and maximal velocity. Moreover, total distance covered with possession of the ball and maximal velocity were the most important variables to predict the total of points obtained at the end of the season. Specifically, the relative contribution of total distance covered with ball possession to the total of points obtained was greater than maximal velocity. Training programs for professional soccer players should be focused on improving the sprint capacity and running with possession of the ball (e.g., transitional tasks and small-sided games). Moreover, this has implications for injury prevention, physical, psychological, and technical-tactical training since today’s soccer requires players to engage in repeated high-intensity actions, reach maximum speeds above 9 m/s, and develop technical-tactical coordination when running with the ball.

## Introduction

Over the last decade, player monitoring in soccer has evolved through the increasing use of electronic performance and tracking systems (Akenhead and Nassis, 2016; Linke et al., 2018; Pino-Ortega et al., 2021). The development of microtechnology embedded in the same tracking unit (e.g., micro electrical mechanical systems and Global Navigation Satellite Systems) allows players’ monitoring based on variables such as the total distance covered, the total of sprinting actions, or the total of impacts experienced by players (Oliva-Lozano et al., 2021a). For instance, information obtained from players’ monitoring may be used for decisions related to the training schedule such as the programming of subsequent training or recovery activities to maximize performance and reduce the injury risk (Oliva-Lozano et al., 2021b).

In this regard, many studies have reported that knowledge of physical demands of competition is necessary to prescribe the optimum training load (Bourdon et al., 2017; Clemente et al., 2019; Oliva-Lozano et al., 2021c). In recent years, an increase in intensity of match running demands has been observed in different European leagues (Barnes et al., 2014; Pons et al., 2021; Rago et al., 2020). Recent studies observed that the players ran ~10-11 km per match in German, Spanish and English professional soccer leagues, being about ~5-7% of the total distance covered at high intensity (Chmura et al., 2019; Oliva-Lozano et al., 2022; Reynolds et al., 2021). Indeed, professional soccer players may cover 200 m/min in the most demanding passages of play (Oliva-Lozano et al., 2020a, 2021c). In this regard, many researchers highlight that players have become faster and perform more frequent sprints in modern soccer (Andrzejewski et al., 2018; Chmura et al., 2017; Haugen et al., 2014; Oliva-Lozano et al., 2020b). Nonetheless, each league may require different physical or technical performance to be successful at the end of the season (Dellal et al., 2011). For instance, English FA Premier League players generally cover greater sprinting distance than LaLiga players.

Furthermore, a previous study found that running with ball possession was significantly associated with team success (i.e., final ranking position in the league based on the total of points obtained) considering that higher-ranked teams cover greater distance in ball possession than lower-ranked teams (Brito Souza et al., 2020). Thus, data of match running performance of players with or without the ball would be of great interest to prescribe sport-specific drills, improve physical performance as well as tactical behavior (Andrzejewski et al., 2014; Riboli et al., 2021). However, the true influence of these match running performance variables on overall soccer success is still unknown (Brito Souza et al., 2020; Del Coso et al., 2020; Hoppe et al., 2015).

The game is constantly changing and from a practical point of view, it is important to provide coaches with information about the physical demands of current soccer (Pons et al., 2021). Also, research is scarce in terms of analyzing the association between match running performance and team success. Therefore, the aims of this study were to: 1) determine the match running performance required by high-ranked, medium-ranked, and low-ranked teams in the German league; and 2) analyze the association between match running performance and teams’ success at the end of the season.

## Methods

### Design and Procedures

A longitudinal study over two consecutive seasons was conducted in the German Bundesliga. Match running performance variables were collected using a vision-based tracking system (VIS.TRACK), which was the official electronic performance and tracking system of the league. Data from all matches during both seasons were analyzed. In addition, the final ranking position and the total of points obtained by each team at the end of the season were registered for the analysis of the association between team success and match running performance.

### Participants

Data were collected from professional soccer players competing in the German Bundesliga during 2017-2018 and 2018-2019 seasons. The league was composed of 18 teams and each team played a total of 34 matches per season, which resulted in 1,224 match observations. This study maintains the anonymity of the players following data protection law. It was conducted in compliance with the Declaration of Helsinki and approved by the local Ethics Committee.

### Measures

The kinematic analysis was carried out through the use of the IMPIRE AG match analysis system (Tiedemann et al., 2011) at a sampling frequency of 25 Hz. IMPIRE AG (Ismaning, Germany) and Cairos Technologies AG (Karlsbad, Germany) provided a ready to use vision-based tracking system for team sports called VIS.TRACK. Each player’s movement was recorded by two cameras (Link and Weber, 2017) to generate positions of players by image recognition technology (Siegle et al., 2013). The system utilized state-of-the-art algorithms and 2-D and 3-D video-recording technology, allowing for detailed motion analysis of players with and without the ball of entire soccer matches. Match events coded by independent operators using this system achieved very good levels of agreement (weighted kappa values of 0.92 and 0.94), with a mean difference of event time equal to 0.06 ± 0.04 s (Liu et al., 2013). The variables, which were reported in each match performance, were included in the dataset.

Specifically, the tracking systems allowed the data collection of the following match running performance variables: total running distance (TD) covered with possession of the ball, TD covered without possession of the ball, sprinting distance (SPD, above 6.3 m/s) covered with possession of the ball, SPD covered without possession of the ball, total of sprinting actions (SPA, above 6.3 m/s) with possession of the ball, SPA without possession of the ball and maximum speed reached in the match (V_MAX_). Specifically, the speed thresholds for sprinting actions were set according to previous studies (Hoppe et al., 2015; Link and de Lorenzo, 2016). Also, the final ranking position at the end of each season was used to categorize the teams into high-ranked teams (from the 1^st^ to the 6^th^ team), medium-ranked teams (from the 7^th^ to the 15^th^ team), and low-ranked teams (from the 16^th^ to the 18^th^ team) (Asian Clemente et al., 2019; Brito Souza et al., 2020). Data from both seasons were included in the same dataset to increase the sample size. This implies that if a team participated in the first season, this team was considered as a different “sample” for the second season. Then, if that team was the 1^st^ team in the first season, its data were categorized as a high-ranked team. However, if that team was the 7^th^ team in the second season, its data were categorized as a medium-ranked team. In addition, the total of points obtained by each team at the end of the season were registered for the analysis of the association between team success and match running performance.

### Statistical Analysis

First of all, means per season and team were calculated for each running performance variable. Then, the Shapiro-Wilk test was used to verify the normality of the variables and all of them followed a normal distribution. Also, homogeneity of variance was assumed since the results of the Levene's test were not significant. Regarding the first aim of the study, descriptive statistics were obtained to show the match running performance required by high-ranked, medium-ranked, and low-ranked teams. In addition, a multivariate analysis of variance (MANOVA) with the Bonferroni post-hoc test was conducted to compare the match running performance between high-ranked, medium-ranked, and low-ranked teams. The Pearson’s correlation coefficient (r) was used to analyze the association between the match running performance variables and teams’ success based on the total of points obtained at the end of the season. Then, match running performance variables which were significantly (*p* < 0.05) associated with the total of points obtained at the end of the season, were included in a multiple linear regression analysis. This regression analysis allowed to assess the influence that match running performance variables had on the total of points obtained at the end of the season based on a stepwise method. Specifically, the standardized regression coefficients showed the relative contribution of each running performance variable to the total of points obtained at the end of the season while the unstandardized coefficients were reported to obtain the equation that estimated the total of points at the end of the season. Effect sizes (ES) were estimated with partial eta-squared. All statistical analyses were conducted using SPSS Statistics version 25 (IBM Corp., Armonk, NY, USA).

## Results

**Figure 1:**
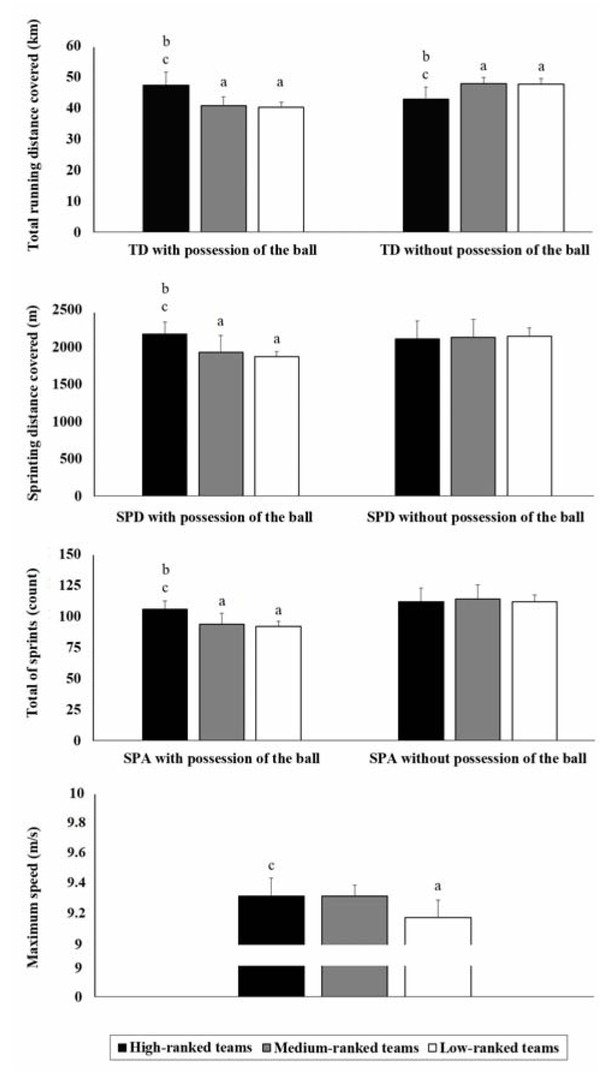
Match running performance (mean ± standard deviation) variables required by high-ranked, medium-ranked, and low-ranked teams in the German League. ^a^: Statistical difference compared to high-ranked teams (p < 0.05). ^b^: Statistical difference compared to medium-ranked teams (p < 0.05). ^c^: Statistical difference compared to low-ranked teams (p < 0.05).

Figure 1 shows match running performance variables by high-ranked, medium-ranked, and low-ranked teams from the German league over two competitive seasons. The ranking position had a significant effect on match running performance variables (F_(14,56)_ = 3.12; *p* < 0.001; ES = 0.45). The results reported a significant interaction between: (a) the ranking position and the TD covered with possession of the ball (F_(2,33)_ = 17.10; *p* < 0.001; ES = 0.51); (b) the ranking position and the SPD covered with possession of the ball (F_(2,33)_ = 7.53; *p* = 0.002; ES = 0.31); (c) the ranking position and the SPA with possession of the ball (F_(2,33)_ = 10.25; *p* < 0.001; ES = 0.38); (d) the ranking position and the TD covered without possession of the ball (F_(2,33)_ = 12.72; *p* < 0.001; ES = 0.44); and (e) the ranking position and V_MAX_ (F_(2,33)_ = 3.97; *p* = 0.03; ES = 0.20).

Specifically, the pairwise comparisons showed that high-ranked teams achieved the greatest TD covered with ball possession (47.24 ± 4.21 km per match), SPD covered with ball possession (2.17 ± 0.16 km per match), and SPA with ball possession (105.52 ± 6.92 actions per match) in addition to V_MAX_ (9.32 ± 0.11 m/s per match). On the contrary, the TD covered without possession of the ball by medium-ranked (47.79 ± 1.99 km per match), and low-ranked teams (47.57 ± 1.95 km per match) was significantly greater (*p* < 0.001; ES = 0.44) than in high-ranked teams (42.79 ± 3.91 km). However, there was no significant interaction between the ranking position and the SPA without possession of the ball (F_(2,33)_ = 0.17; *p* = 0.84; ES = 0.01) or the SPD without possession of the ball (F_(2,33)_ = 0.06; *p* = 0.95; ES = 0.01).

Regarding the second aim of the study, a significant correlation (r = 0.4-0.7; *p* < 0.01) between the team success (i.e., total of points obtained at the end of the season) and all match running performance variables was observed, except for SPD (r = -0.12; *p* = 0.48) and SPA (r=-0.11; *p* = 0.51) without possession of the ball. However, the multiple linear regression model showed that variables that significantly contributed to the model were TD covered with possession of the ball and V_MAX_ (Table 1), which explained 70% of the variance in the total of points obtained at the end of the season (R^2^ = 0.7). Specifically, the relative contribution of TD covered with possession of the ball to the total of points obtained at the end of the season (Beta = 0.72) was greater than V_MAX_ (Beta = 0.36). In this regard, the equation to predict the total of points obtained at the end of the season was:

**Table 1:** Standardized and unstandardized coefficients from the multiple linear regression model

Model	Unstandardized coefficients	Standardized coefficients	95% CI	
	B SE	Beta	Min	Max
Constant	-545.14 131.50		-812.68	-277.6
TD covered with ball possession	2.51 0.34	0.72*	1.82	3.21
V^MAX^	14.50 3.94	0.36*	6.47	22.54

***Note*** : SE = Standard error; CI = Confidence interval; Min = Minimum value; Max = Maximum value; TD = Total running distance; V_MAX_ = Maximum speed; *p-value < 0.05

**Points = -545.14 (i.e., B constant) + 2.51 × TD covered with ball possession + 14.50 × V_MAX_**.

## Discussion

The aims of this study were to analyze match running performance required by teams based on their final league ranking position and to determine the association between match running performance and teams’ success at the end of the seasons analyzed. The main findings were that there was a significant interaction between the ranking position and the TD, SPD, and SPA with possession of the ball. In addition, the TD covered without possession of the ball and V_MAX_ reached in match play had a significant interaction with the ranking position. Specifically, the TD covered with possession of the ball and V_MAX_ were the most important variables to predict the final ranking position at the end of the season in a professional soccer league over two competitive seasons.

Previous research showed that higher-ranked teams ran greater distance with ball possession than lower-ranked teams (Brito Souza et al., 2020). Also, it was observed that sprinting actions were associated with an increased probability to achieve the first positions of the final ranking in the Italian Serie A (Longo et al., 2019). Our research not only confirms these findings, but also suggests that SPD and SPA with possession of the ball are significant factors of reaching a higher position in the ranking at the end of the season. This implies that teams do not only need to run with the ball, but also run with the ball at high intensity (Brito Souza et al., 2020). This thesis is also supported by the second result of this study regarding V_MAX_, although this variable with/without ball possession was not recorded due to tracking system limitations. For instance, a recent study showed that 94.3% of professional soccer players may run above 8.3 m/s in Spanish LaLiga matches (Del Coso et al., 2020; Oliva-Lozano et al., 2021d) and some players registered 9.7 m/s (Del Coso et al., 2020), but these actions are quite unique during the course of a game (Oliva-Lozano, Fortes, et al., 2021d). In consequence, this may benefit players when scoring a goal since straight sprinting actions are the most frequent actions in goal situations and running speed is key for overtaking opponents and winning disputed balls (Faude et al., 2012). However, another investigation conducted in the Spanish LaLiga showed that both higher- and lower-ranked teams presented the same running requirements at high speeds (i.e., above 5.8 m/s). Also, the same study found similar running distance with ball possession in teams classified for the Champions League and teams that relegated to an inferior division (Asian Clemente et al., 2019).

In addition, one of the greatest challenges of modern performance analysis is to find variables that positively influence a team's end-of-season success (Konefał et al., 2019a). For instance, the running distance and the number of activities performed with ball possession were positively associated with total of points at the end of the season (Hoppe et al., 2015).

Specifically, these authors observed that the running distance covered with ball possession explained 60% of the variance in the final points. This is consistent with our results since TD covered with possession of the ball and V_MAX_ explained 70% of the variance in the total of points obtained at the end of the season. In this regard, the coefficient of determination (R^2^ = 0.7) obtained by the multiple linear regression model was very high taking into account the complexity of the game and the multitude factors influencing the modern game (Liu et al., 2016). Nonetheless, the results also highlighted the importance of TD covered with possession of the ball since it was the most significant variable to predict the total of points obtained (Beta = 0.72). Specifically, running with the ball increases physiological stress compared to regular running (Carling, 2010). Then, additional energy expenditure is required for this match activity, which should be considered when evaluating physical performance. Therefore, given the role of TD covered with ball possession and V_MAX_ reached in the team success at the end of the season, this demonstrates that current soccer players need to be prepared for high physical and technical demands in addition to tactical and psychological demands required at a professional level.

The authors are fully aware of the many factors that might have influenced the results of the present study. For example, a limitation is that research should be developed considering the influence of different contextual variables (e.g., playing position, length of the microcycle, match location, or match outcome) (Konefał et al., 2019b; Oliva-Lozano et al., 2020c). In addition, the present study was focused on a specific league (i.e., the Bundesliga), and therefore, the data obtained should be interpreted with caution. Future investigations with a more holistic approach should include not only physical performance, but also psychological, tactical, and technical performance (e.g., total of successful and unsuccessful passes, total of shots on goal, attempted dribbles, etc.).

In summary, teams that cover greater running distance with ball possession and teams with maximum sprinting speeds have greater success at the end of the season in a professional soccer league in Germany. Moreover, TD covered with possession of the ball and V_MAX_ were the most important variables to predict the total of points obtained at the end of the season. These findings may provide insights into the establishment of team performance profiles with the development of specific training drills, which may optimize the playing style, although future investigations should consider the effect of a playing position.

In conclusion, professional soccer players in the German league experience high match running demands. This suggests that high levels of aerobic capacity, tolerance to increasing fatigue, and anaerobic capacity may be required at this level. Training programs for professional soccer players should be focused on improving the sprint capacity and running with possession of the ball. For example, this may be trained by applying repeated transitions (i.e., involving players in offensive actions followed by defensive actions, and vice versa) and small-sided games. In addition, this has implications not only for physical training, but also for psychological and technical-tactical preparation since today’s soccer demands require players to engage in repeated high-intensity actions, reach maximum speeds above 9 m/s, and develop both technical coordination and tactical behavior when running with the ball.
